# Fatty Acid Supplementation During *in vitro* Embryo Production Determines Cryosurvival Characteristics of Bovine Blastocysts

**DOI:** 10.3389/fcell.2022.837405

**Published:** 2022-03-09

**Authors:** H. Aardema, I. Bertijn, HTA. van Tol, A. Rijneveld, JCM. Vernooij, B. M. Gadella, PLAM Vos

**Affiliations:** Department of Population Health Sciences - Farm Animal Health, Faculty of Veterinary Medicine, Utrecht University, Utrecht, Netherlands

**Keywords:** free fatty acid, embryo, blastocyst, cryosurvival, lipid, apoptose, slow freezing, cryopreservation

## Abstract

*In vitro* production (IVP) embryos have a reduced quality and poor cryotolerance in comparison to *in vivo* embryos. This study investigated whether free fatty acid (FFA) conditions, fatty acid free (FAF)- synthetic oviduct fluid (SOF) without or with 25 μM of saturated stearic (C_18:0_) or unsaturated oleic (C_18:1_) acid during the first 5 IVP days, relate to quality and cryosurvival of day 8 blastocysts. Apart from the blastocyst scores, both 1) number and size of lipid droplets of fresh blastocysts and 2) total number and apoptotic and necrotic cells, before and after freezing-thawing, were scored by confocal microscopy. Blastocyst rates were significantly lower in the FAF SOF condition in comparison to other groups. Interestingly, blastocysts originating from the C_18:1_ group, with a significantly higher lipid content, and blastocysts from the FAF SOF group demonstrated a high cryosurvival rate (70.1 and 67.4%, respectively) comparable with *in vivo* blastocysts (68%), in contrast to the poor cryosurvival of C_18:0_ exposed embryos (17.6%). In all freeze-thawed embryos the average amount of apoptotic and necrotic cells increased albeit that the C_18:0_ condition rates were higher (43.2%) when compared to C_18:1_ (26.0%) and FAF SOF conditions (26.5%). The current data show that FFA administered during early embryonic development significantly affect the cryotolerance of blastocysts.

## 1 Introduction

Worldwide, the number of *in vitro* production (IVP) bovine embryos has dramatically increased during the last years, until more than 1 million in 2019 with a concomitant decrease in the number of *in vivo* derived embryos (378.769 in 2019; (Viana, 2020)). Genomic selection technologies performed by breeding companies have increased the demand for IVP produced embryos, derived from genetically desired animals, to shorten the generation intervals and to increase the genomic gain. Harvesting oocytes by ovum pick up (OPU) offers the possibility to retrieve a higher number of IVP embryos per individual animal over time in comparison to the technology of multiple ovulation embryo transfer (MOET), which is related with high donor variability producing unpredictable numbers of *in vivo* embryos (Boland et al., 1991; Hasler et al., 2003; Ferré et al., 2020). To this end, it is not surprising that in 2016 worldwide the number of IVP embryos for the first time exceeded the number of *in vivo* derived embryos (Viana, 2020). In order to manage these high numbers of embryos, cryopreservation is an ideal tool for logistical reasons because breeding companies can optimally time the transfer of the embryo with the reproductive cycle of the recipient and transfer the embryo over long distances, but unfortunately the cryosurvival rates of IVP embryos are low in comparison to *in vivo* derived embryos (Moore and Hasler 2017; Viana, 2020; Ferré et al., 2020). However, the currently still reduced cryosurvival of IVP embryos versus *in vivo* derived embryos is costly and puts a significant burden on bovine industry. Therefore, optimization of the IVP conditions in order to increase the cryosurvival of IVP embryos is highly desirable.

It is well known that the potential of an oocyte to develop into a blastocyst after *in vitro* fertilization and subsequent embryo culture is largely depending on the quality of the oocyte, with only 30–40% of the *in vitro* matured oocytes reaching the blastocyst stage in contrast to 80% of the *in vivo* matured oocytes (Hendriksen et al., 2000; Dieleman et al., 2002; Rizos et al., 2002; Knijn et al., 2003; Tesfaye et al., 2007). However, the quality of the embryo itself and thus the options for cryosurvival can be improved by optimizing the post-fertilization conditions of IVP embryos (Rizos et al., 2002; Lonergan 2007; Lonergan and Fair 2008; Gad et al., 2012).

A higher lipid content in IVP embryos has been suggested as one of the factors for decreased quality and cryosurvival rates when compared to *in vivo* derived embryos (Abe et al., 2002; Hara et al., 2005; Reis et al., 2005; Pereira et al., 2007; Sudano et al., 2011; Abe et al., 2017). Moreover, the lipid composition in embryos has also been described as one of the possible factors causing a reduction in the cryosurvival of IVP embryos. In fact high quality oocytes and embryos were shown to contain relatively high proportions of unsaturated fatty acids, linoleic and oleic acid, and low proportions of saturated fatty acids in comparison to oocytes and embryos of lower quality (Hochi et al., 1999; Kim et al., 2001; Haggarty et al., 2006). Furthermore, embryos have been shown to take up exogenous free fatty acid (FFA) during IVP and are able to incorporate these fatty acids in neutral lipid stored in lipid droplets (Flynn and Hillman 1980; Wang and Tsujii 1998; Khandoker and Tsujii 1999; Haggarty et al., 2006; Sturmey et al., 2009; Hughes et al., 2011; McKeegan and Sturmey 2012). Thus in the current study the aim was to investigate whether the quality of IVP embryos and their cryosurvival rates is influenced by supplementation of FFA during the first 5 days of embryonic development, i.e. at the physiological period that the embryo *in vivo* would reside in the oviduct. To this end, presumed zygotes were exposed to stearic acid (C_18:0_) or oleic acid (C_18:1_) during day 1 till 5 of IVP (day 0 = IVF). In literature, both cytoplasm lipid content and cell apoptosis rates have been considered to be important factors that relate to embryo cryosurvival (Sudano et al., 2014). Therefore, the number and size of lipid droplets was investigated in day 8 blastocysts before freezing and the number of apoptotic and necrotic cells in blastocysts before and after freeze/thawing.

## 2 Materials and Methods

### 2.1 Chemicals and Media

All chemicals were obtained from Sigma-Aldrich (Sigma-Aldrich Corp., St. Louis, MO, United States) unless otherwise stated. All culture media were prepared manually, corrected for pH (pH = 7.35 ± 0.05) and filter sterilized through a 0.22 µm Millex GV filter (Merck, Darmstadt, Germany).

### 2.2 Experimental Design

**SCHEME 1:**
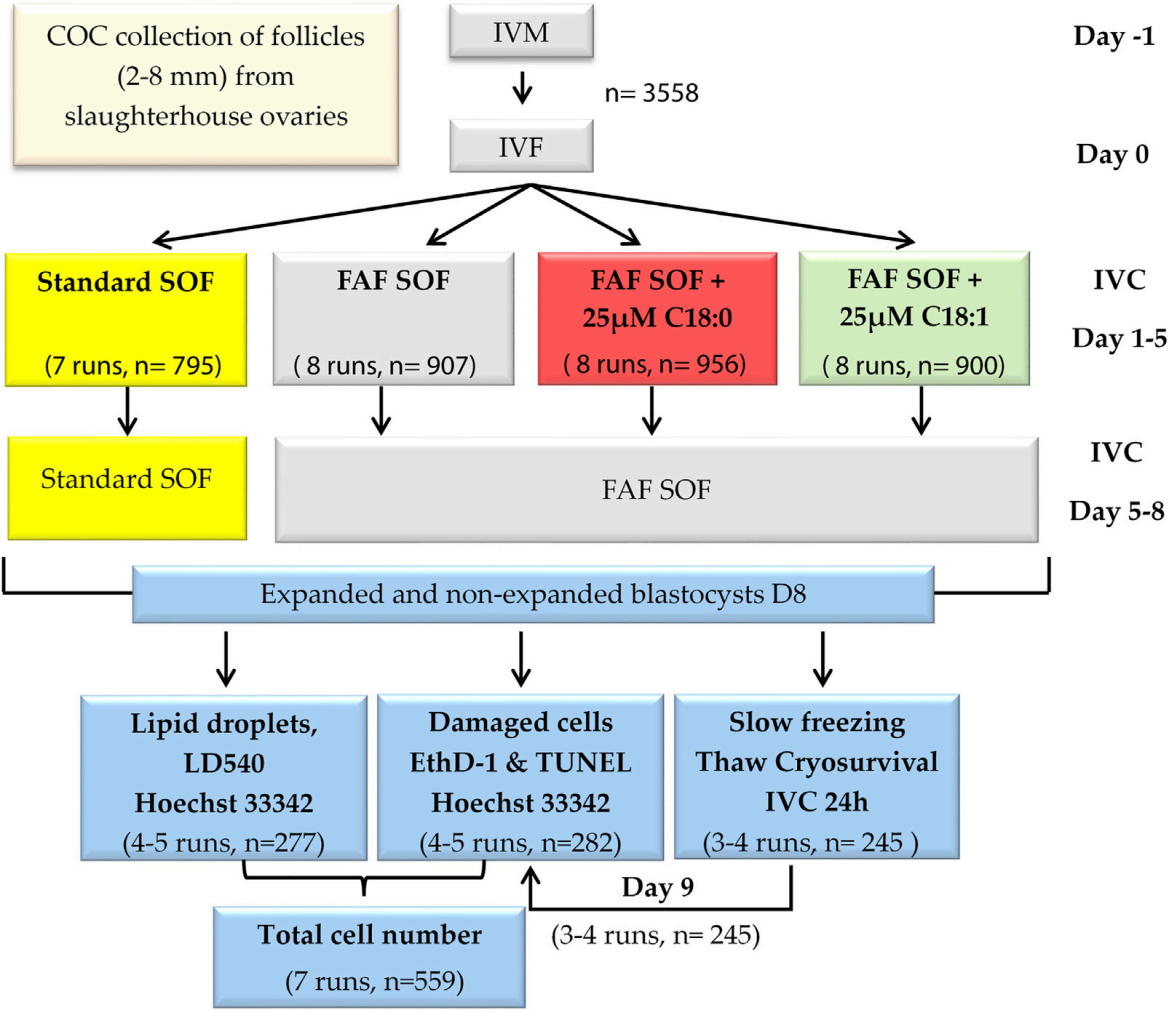
Experimental design. Bovine cumulus oocyte complexes were *in vitro* matured (day -1) and fertilized (day 0). Presumptive zygotes (day 1) were divided into four groups and cultured in synthetic oviduct fluid (SOF) with (i) bovine serum albumin (BSA), Standard SOF (ii) delipidified >96% fatty acid free (FAF) BSA, FAF SOF (iii), FAF BSA complexed with 25 µM unsaturated oleic acid, C_18:1_ or (iv) with saturated stearic acid, C_18:0_.8 days post fertilization, blastocysts were collected and stained for lipid droplets, apoptosis and necrosis or cryopreserved (slow freezing procedure). Frozen-thawed blastocysts were stained for apoptosis and necrosis.

In experiment 1, the effect of fatty acid supplementation during the first 5 days of *in vitro* embryonic development on bovine embryo lipid content, cell damage and cryosurvival was investigated. After standard *in vitro* maturation of cumulus-oocyte-complexes (COCs; day -1), and fertilization (day 0; (Aardema et al., 2011)), presumed zygotes were cultured from day 1 until day 5 in SOF with 1) bovine serum albumin (BSA) (standard SOF) or 2) delipidified BSA (>96% fatty acid free (FAF; FAF SOF) (iii), FAF BSA complexed with 25 µM saturated C_18:0_ or 4) with unsaturated C_18:1_. From day 5 onwards, SOF was refreshed and embryos were cultured in standard embryo culture conditions with BSA (standard SOF) or with delipidified BSA (FAF SOF). At day 8 post-fertilization, fresh and frozen-thawed blastocysts were stained for apoptosis (TUNEL; Roche Cell Death Detection Kit, Sigma Chemical Co., St Louis, MO, United States), necrosis (Ethidium-homodimer 1; EthD-1, Invitrogen, Waltham, MA, United States) and DNA (Hoechst 33342). Another set of fresh blastocysts was fixed in 1% PFA and stained with LD540 (a dye based on the Bodipy fluorophore, fabricated by (Spandl et al., 2009) for lipid droplet (LD) staining. Scheme 1.

### 2.3 *In vitro* Embryo Production

#### 2.3.1 Maturation

*In vitro* embryo production was performed as described previously (Aardema et al., 2011). In short, bovine ovaries were collected from a slaughterhouse and transported to the laboratory within 2 h. Ethical approval was not necessary, as the ovaries were a waste product from regular slaughter. COCs were aspirated from antral follicles (2–8 mm diameter) using a winged infusion set (18 gauge needle) connected to a vacuum suction system (−0.6 bar), pooled in a conical tube and allowed to sediment for 30 min at room temperature. COCs with a minimum of two cumulus cell layers were selected and washed 3x in HEPES buffered M199 cell culture medium with 2.2 mg/ml NaHCO_3_ at room temperature. 50–55 COCs were matured in 500 µL M199 cell culture medium per well with 2.2 mg/ml NaHCO_3_, 0.05 IU/ml Human recombinant follicle-stimulating hormone, 0.1 μM cysteamine, 10 ng/ml epidermal growth factor in 0.1% (w/v) FAF BSA and 1% (v/v) penicillin-streptomycin (Gibco BRL, United States), and no coverage of oil, in four-well culture plates (Nunc A/S, Roskilde, Denmark) for 23 h at 38.5°C in a humidified atmosphere of 5% (v/v) CO_2_ in air, according to our standard protocol (Aardema et al., 2011).

#### 2.3.2 Fertilization

After 23 h of maturation (day -1), *in vitro* fertilization (day 0) was performed with 1 × 10^6^/ml sperm from a bull with proven fertility. Procedures for *in vitro* fertilization were performed as described by Parrish et al. (Parrish et al., 1988) with minor modifications (Izadyar et al., 1996). Briefly, sperm cells were added to the fertilization medium (modified Tyrode’s medium also called Fert-TALP (Parrish et al., 1988), without glucose and 1% (v/v) penicillin-streptomycin instead of gentamycin (Izadyar et al., 1996) to a final concentration of 1 × 10^6^ sperm cells/mL in the presence of 10 μg/ml heparin, 20 μM d-penicillamine, 10 µM hypotaurine, and 1 µM epinephrine. Fertilization lasted for 18–20 h at 38.5°C in a humidified atmosphere of 5% (v/v) CO_2_ in air.

#### 2.3.3 Embryo Culture

At 18–20 h after sperm addition, cumulus cells of presumptive zygotes were removed by vortexing for 3 min. Denuded zygotes were transferred in groups of 50–55 to wells with 500 μL standard SOF or FAF SOF without or with supplementation of 25 µM C_18:0_ or C_18:1_ and cultured at 38.5°C in a humidified atmosphere of 5% (v/v) CO_2_ and 7% (v/v) O_2_. C_18:0_ or C_18:1_ were complexed to >99.9% delipidified bovine serum albumin (BSA), according to our protocol with a fatty acid:BSA stoichiometry of 5:1 (Aardema et al., 2011). On day 5 post insemination, cleavage rates were determined and non-cleaved embryos were discarded. The cleaved embryos were transferred to fresh standard SOF or FAF SOF medium. Blastocyst rates and quality, according to the International Embryo Technology Society (IETS) identification system (Stringfellow and Givens, 2009), were determined at day 7 and 8 post insemination. Blastocysts were collected on day 8 post fertilization for further analysis. Scheme 1.

### 2.4 Cryopreservation

#### 2.4.1 Slow Freezing

On day 8 post insemination, excellent (grade 1) and fair (grade 2) quality blastocysts were selected for the slow freezing procedure, collected in small droplets of standard SOF or FAF SOF and allowed to cool to room temperature in 5 min. Subsequently, they were washed 5 times in EMCARE™ holding solution (ICPbio Reproduction, Glenfield, New Zealand), supplemented with 20 ml/L of 20% (w/v) albumin solution (ICPbio Reproduction, Glenfield, New Zealand), submerged in ViGRO™ Ethylene Glycol Freeze Plus sucrose medium (Vetoquinol SA, Lure, France), loaded onto 0.25 ml embryo transfer straws, and dropped into a -7°C alcohol bath slow freezer (Julabo FP40-ME, Boven-Leeuwen, Netherlands) within 10–20 min after introduction into the freezing medium. Each straw was loaded with 1-2 blastocysts. Straws were seeded after 5 min and held at −7°C for 10 min to complete ice crystal formation. The blastocysts were cooled with a rate of 0.5°C/min until −35°C and plunged into liquid N_2_ at −196°C.

#### 2.4.2 Thawing

Straws with frozen blastocysts were collected from liquid N_2_ and held for 8 s in air, before being plunged into a 22°C water bath for 20 s. Thawed blastocysts were collected in a Petri dish. They were guided through five washing steps at room temperature, consisting of standard SOF or FAF SOF mixed with subsequently 80, 60, 40, 20 and 0% freezing medium, each washing step lasted 5 min. Blastocysts were cultured in standard SOF or FAF SOF to determine the cryosurvival, defined as re-inflation of the blastocoel 24 h post-thawing (Dinnyés et al., 1999; Sudano et al., 2011). *In vivo* derived blastocysts, cryopreserved via a comparable slow freezing procedure, were generously donated by the breeding company CRV (CRV, Arnhem, Netherlands). The *in vivo* derived blastocysts were thawed and cultured for 24 h in standard SOF according to a comparable protocol as described above, to compare the cryosurvival rate of *in vivo* derived blastocysts with the *in vitro* blastocysts derived from the different embryo culture conditions in the current study.

### 2.5 Apoptosis and Necrosis Staining

Necrosis and late-stage apoptosis were detected with EthD1 and a TUNEL assay, respectively, as described by Pomar et al. (Pomar et al., 2004), with minor modifications. Briefly, blastocysts were incubated in 4 μM EthD-1 in PBS for 5 min in the dark at room temperature and fixed in 4% w/v paraformaldehyde (PFA) overnight (J.T. Baker Chemicals Co., Phillipsburg, NJ, United States). Thereafter, blastocysts were permeabilized in PBS with 0.1 vol% Triton X-100 and 0.1% w/v sodium citrate for 5 min on ice, incubated in TUNEL reaction mixture for 1 h at 37°C in the dark and subsequently stained with 10 μM Hoechst 33342 in PBS for 30 min. Blastocysts were mounted on glass slides between 120 μm spacers in 100% Vectashield (Vector Laboratories Inc., Burlingame, CA, United States). Before and after each staining step, blastocysts were washed three times in 0.3% w/v polyvinylpyrrolidone (PVP)-PBS for 3 min. As a positive control, several blastocysts were exposed to DNAse (0.0027 Units, diluted in 50 mM TRIS buffer, pH 7.5 with 1 mg/ml BSA, RNase free DNase set, Qiagen, Hilden, Germany) for 10 min before incubating in TUNEL reaction mixture. Negative controls were created by incubating blastocysts in TUNEL Label solution.

### 2.6 Lipid Staining

Neutral lipid droplets were stained using LD540, a dye based on the Bodipy fluorophore, fabricated by (Spandl et al., 2009). Blastocysts were washed once in 0.3% (w/v) PVP-PBS, fixed at room temperature in 4% w/v PFA solution (J.T. Baker Chemicals Co., Phillipsburg, NJ, United States) for 20 min and stored in 1% w/v PFA at 4°C. Fixed blastocysts were incubated in 10 μg/ml Hoechst 33342 and 0.1 μg/ml LD540 in PBS for 30 min in the dark at room temperature. Before and after staining, blastocysts were washed 3x in 0.3% (w/v) PVP-PBS- for 3 min and subsequently mounted on glass slides between 120 μm spacers in 100% Vectashield (Vector Laboratories Inc., Burlingame, CA, United States).

### 2.7 Fluorescent Imaging

Confocal microscopy was performed using a Leica SPE-II–DM14000 laser scanning microscope (Leica microsystems, Wetzlar, Germany), using a ×20 magnification oil immersion objective combined with 1.5 digital zoom, resulting in ×30 magnification. Leica LAS software was used for acquiring images (LAS-AF, HCS Basic Module, Leica microsystems, Wetzlar, Germany). Blastocysts were imaged using z-stack serial sections with 10 µm steps and were excited sequentially with 405 nm, 488 and 561 nm at 7–14% laser strength for the detection of Hoechst 33342, TUNEL and EthD-1, respectively. Emission was detected at 420–490 nm, 500–550 nm and 580–650 nm, respectively. The LD540 stain was imaged using 488 nm excitation and 500–550 nm emission. Differential interference contrast (DIC) images were made using 488 nm excitation and 500–550 nm emission. The 8-bit z-stack series were analyzed using FIJI (Schindelin et al., 2012); ImageJ 1.48t, Java 6.0_24). LD540 stained lipid droplets were counted using a macro with the following steps: subtract background (“rolling = 10”), set threshold (55–125 over 255) per run, watershed, count particles (>0.3 µm to avoid counting background noise). The total number of droplets, average droplet area (in µm^2^) and standard deviation per blastocyst were documented. The number of nuclei per blastocyst (Hoechst 33342 staining) was counted according to the following procedure; 1) enhance contrast (0.5%), 2) set threshold (45 over 255), 3) fill holes, 4) watershed, 5) count particles (>50 µm with a minimal circularity of 0.2). Necrotic and apoptotic positive cells were counted manually in each separate z-stack image using the LAS AF Lite software (LAS AF lite, version 2.6.3, Leica microsystems, Wetzlar, Germany). A nucleus was scored as necrotic when the EthD-1 stain was visible and corresponded to an Hoechst 33342 stained nucleus in the overlay image. Apoptotic cells were counted similarly, using the TUNEL stain.

### 2.8 Statistical Analysis

Cleavage score (total cleaved/oocytes), blastocyst score day 7 and 8 (total blastocysts/oocytes), and cryosurvival, defined as expansion of the blastocoel 24 h post-thawing (re-expanded blastocysts/total thawed), were analyzed by mixed effect grouped binomial logistic regression models with experimental group as explanatory variable. For cryosurvival, the explanatory variable blastocyst development stage and its interaction with the experimental group was also tested. The number of lipid droplets and nuclei, respectively, were analyzed by a mixed effect regression model with a Poisson link. Average lipid droplet size, number and total lipid (average lipid droplet size * number of lipid droplets) were analyzed with a mixed effect linear regression model. Residuals of the fitted model assumptions were visually inspected and therefore both outcome variables were log transformed to meet the assumptions. Experimental group was added as explanatory variable. For the model that analyses the number of nuclei per blastocyst, explanatory variable blastocyst development stage and its interaction with the experimental group was tested. The proportion of apoptotic-, necrotic- and double stained cells per blastocyst (stained with TUNEL and/or EthD) were analyzed like binomial logistic regression models. Besides the explanatory variable of experimental group also the variable thaw/fresh and the interaction between group and thaw/fresh was added to the model. In all models, run (replicate number of the experiment) was added as random effect to account for the correlation of observations within each run. A second random effect was added in the models testing cleavage score, day 7 and 8 blastocyst score and cryosurvival to account for the grouping of oocytes or blastocysts in these models. The group and blastocyst development stage effects were presented as odds ratio (OR) with 95% confidence interval (CI) for the binomial logistic regression models, ratio’s for the models with Poisson link and ratio’s between means for the linear regression models due to the log-transformation of the outcome variables. Pairwise comparison between groups were made using Tukey HSD. Blastocyst developmental stages were compared within each experimental group using a reference developmental stage. The Akaike’s Information Criterion (AIC) was used to select the best fitting model but variable experiment group remained in the model at all times as this answered the research questions. The models were fitted in statistical software R version 4.0.5 (R Core Team, 2021, (R Core Team 2021)) with library lme4 (Bates et al., 2015) and multcomp (Hothorn et al., 2008). Graphs were made using library ggplot2 (Wickham 2016) and dplyr (Wickham et al., 2020). Group differences were based on the 95% CI. When the 95% CI of the groups overlapped there was no significant difference (OR = 1). When the 95% CI were different, a higher or lower value gave information about the direction of the difference (increased or decreased). Furthermore, the width of the 95% CI shows the range and precision of the group estimate.

## 3 Results

### 3.1 A Fatty Acid Free Condition Results in Reduced Embryo Development

**FIGURE 1:**
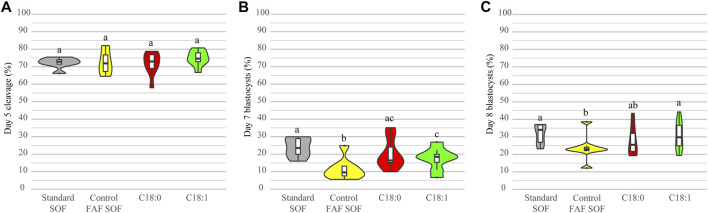
Developmental competence after exposure to different conditions. Bovine embryos were exposed to standard SOF, control FAF SOF, 25 μM C_18:0_ or 25 μM C_18:1_ during day 1 until 5 of *in vitro* culture. The total cleavage was scored at day 5 of embryo culture **(A)** and blastocyst rates were scored at day 7 **(B)** and 8 **(C)** of embryo culture. Different superscripts differ significantly. For runs and total number see Scheme 1 (in the Materials and Methods section).

Presumed zygotes were exposed to either C_18:1_ or C_18:0_ from day 1 until day 5 of embryo culture (day 0 = IVF), in order to investigate whether these FFA affect embryonic development. The control group was cultured in the same fatty acid free synthetic oviduct fluid (FAF SOF) medium without the addition of FFA. The standard (non-FAF) SOF was used as an internal control for the embryo culture procedure. The cleavage rates at day 5 were comparable among the groups (Figure 1A). On day 7 there was a significantly lower blastocyst rate in the FAF SOF group (Figure 1B) in comparison to all groups; odds ratio (OR) 0.41, 0.50 and 0.64 with 95% confidence interval (CI) 0.29–0.58, 0.36–0.69 and 0.46–0.89, for standard SOF, C_18:0_ and C_18:1_ groups respectively. There was a significantly lower blastocyst rate on day 8 for embryos exposed to a FAF SOF (23.7 ± 7.2%) in comparison to those exposed to a standard (non-FAF) SOF (31.7 ± 5.9%; OR 0.66, 95% CI 0.48–0.90) and C_18:1_ condition (30.8 ± 8.4%; OR 0.73, 95% CI 0.54–0.98). On day 8, the blastocyst yield of embryos exposed to FAF SOF was comparable with the C_18:0_ condition (27.9 ± 8.6%; OR 0.77, 95% CI 0.57–1.04, Figure 1C). Note that FAF SOF medium is a chemical defined medium for embryo culture which yields lower blastocyst rates at day 8 when compared to SOF with the addition of fetal calf serum (FCS). Experimentally, the effects of addion of FA to SOF can only be tested in chemical defined medium without FCS and compared to FAF SOF.

### 3.2 Free Fatty Acid Exposure has Minor Effects on the Cell Number in Blastocysts

**FIGURE 2:**
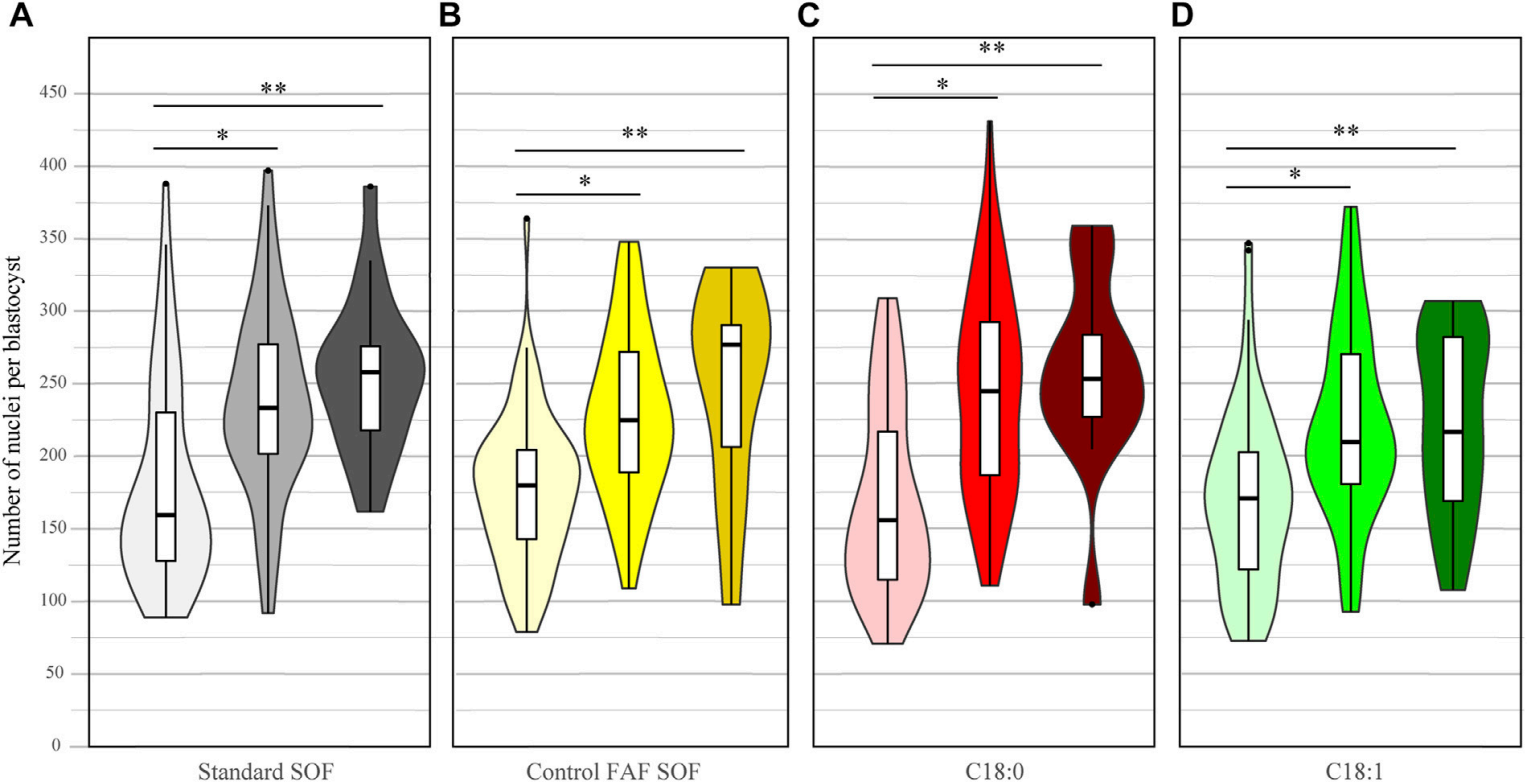
Number of nuclei per day 8 blastocyst. Bovine embryos were exposed to standard SOF **(A)**, control FAF SOF (B), 25 μM C_18:0_ **(C)** or 25 μM C_18:1_ **(D)** during day 1 until 5 of *in vitro* culture. The figure presents the number of nuclei per non-expanded (light), expanded (bright) and hatched (dark) blastocyst per group. Nuclei were stained by Hoechst 33342. Asterix indicates a significant difference between the blastocysts classes within a group. For runs and total number see Scheme 1 (in the Materials and Methods section).

Likewise, the total cell number of blastocysts was scored for all IVP embryo groups at day 8. There was a significant difference among the number of cells per blastocyst between FAF SOF and the other groups, but biological differences in the total number of cells were small (OR 0.96 CI 0.94–0.98 and OR 1.03 CI 1.01–1.04, for C_18:0_ and C_18:1_, respectively; data not shown). This finding indicates that the different FFA exposures had a minor effect on the blastocyst size at day 8 of IVP. In contrast, the blastocyst stage did have a clear effect on the number of cells. Within all groups, non-expanded blastocysts had a significantly lower nuclei number compared to expanded blastocysts (ratio 0.75, 0.76, 0.68 and 0.71, 95% CI 0.73–0.77, 0.74–0.78, 0.67–0.70 and 0.70–0.73 for respectively standard SOF, FAF SOF, C_18:0_ and C_18:1_), and hatched blastocysts (ratio 0.69, 0.74, 0.65 and 0.73, 95% CI 0.67–0.72, 0.71–0.77, 0.62–0.67 and 0.71–0.76 for respectively standard SOF, FAF SOF, C_18:0_ and C_18:1_). Figure 2.

### 3.3 C_18:1_ Exposure Results in Higher Lipid Content of Blastocysts

**FIGURE 3:**
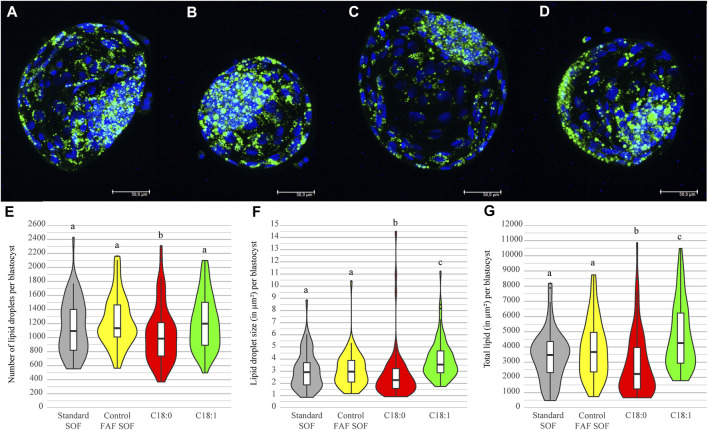
Lipid droplet number and size per blastocyst. Bovine embryos were exposed to standard SOF **(A)**, control FAFSOF **(B)**, 25 μM C_18:0_ **(C)** or 25 μM C_18:1_ **(D)** during day 1 until 5 of *in vitro* culture. Panel A–D shows representative images for the different groups with in blue DNA (Hoechst 33342) and in green lipid droplets (LD540), Bar = 50 µm. Lipid droplet number **(E)**, size **(F)** and total lipid in μm^2^ **(G)** are shown for blastocysts of each group. Different superscripts differ significantly. For runs and total number see Scheme 1 (in the Materials and Methods section).

In order to test whether the lipid storage of IVP embryos can be influenced by FFA exposure during the first 5 days of embryo culture, the amount and size of cytoplasmic lipid droplets was determined from day 8 blastocysts in each exposure group. The number of lipid droplets in embryos exposed to C_18:0_ was significantly lower (1043.79 ± 406.70) than in the FAF SOF and C_18:1_ group (ratio 0.83 and 0.86, 95% CI 0.83–0.84 and 0.86–0.87, resp.; Figure 3E). Interestingly, blastocysts of embryos exposed to C_18:1_ demonstrated a larger lipid droplet size (3.96 ± 1.62 μm^2^), measured two dimensionally by confocal microscopy, than the FAF SOF (3.11 ± 1.44 μm^2^, ratio 1.33, 95% CI 1.15–1.54) and the C_18:0_ (2.70 ± 2.16 μm^2^, ratio 1.63 95% CI 1.41–1.88) group (Figure 3F). Over all, blastocysts exposed to C_18:1_ contained significantly more lipid (lipid droplet number x size) than blastocysts from the other groups (ratio 1.27 and 1.93 and 95% CI 1.05–1.54 and 1.60–2.32, compared to FAF SOF and C_18:0_ resp.; Figure 3G). In contrast, blastocysts of the C_18:0_ exposed embryos had a lower total lipid amount than those of the FAF SOF group (ratio 0.76, 95% CI 0.62–0.93).

### 3.4 Cryopreservation Results in High Level of Damaged Cells in C_18:0_ Blastocysts

**FIGURE 4:**
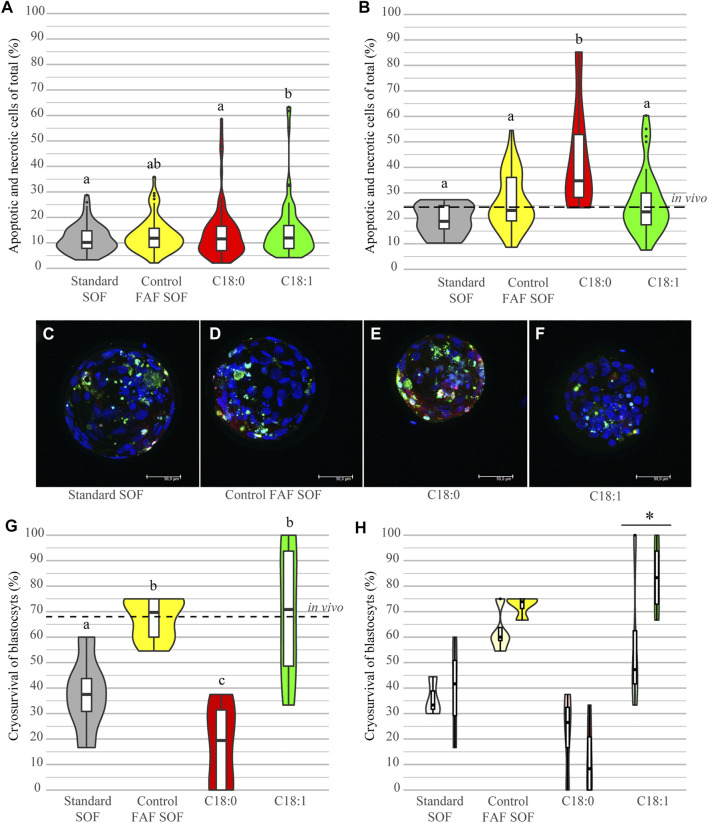
Cell damage and cryosurvival of blastocysts. Bovine embryos were exposed to standard SOF, control FAF SOF, 25 μM C_18:0_ or 25 μM C_18:1_ during day 1 until 5 of *in vitro* culture. The percentage of damaged cells reflects the total sum of apoptotic (TUNEL) and necrotic (EthD-1) cells, from fresh blastocysts **(A)** and those that survived the freezing and thawing procedure **(B)** as a percentage of the total cell number (Hoechst 33342), analyzed via confocal microscopy. A representative confocal image of a frozen thawed blastocyst per group is shown for Standard SOF **(C)**, FAF SOF **(D)**, C_18:0_ **(D)** and C_18:1_ **(E)**, with in blue DNA (Hoechst 33342), in green apoptosis (TUNEL) and necrosis in red (EthD-1). Panel G shows the cryosurvival rates per group. Panel H shows the cryosurvival rates per for respectively non-expanded and expanded blastocysts per group. Bar = 50 µm. Different superscripts differ significantly. Asterix indicates a significant difference between the blastocysts classes within a group. For runs and total number see Scheme 1 (in the Materials and Methods section).

To investigate the impact of cryopreservation on cell survival, the amount of apoptotic (TUNEL) and necrotic (EthD-1) cells in fresh and frozen-thawed blastocysts was scored. Fresh, non-frozen, day 8 blastocysts from the different groups demonstrated a low level of cell damage. The highest level of damaged cells was observed in the group of C_18:1_ (14.92 ± 11.52%) versus the standard SOF (11.56 ± 5.48%; OR 1.15, 95% CI 1.07–1.24), C_18:0_ (13.93 ± 10.88%; OR 1.16, 95% CI 1.09–1.25) and control FAF SOF (13.03 ± 6.73%, OR 1.07, 95% CI 1.00–1.16), resulting in small differences among the groups and significant changes, but these were not considered to be biologically relevant (Figure 4A). The analysis of the damaged cells post freeze-thawing (Figure 4B) showed for all groups a significant increase in the percentage of apoptotic and necrotic cells compared to fresh blastocysts (Figure 4A). Remarkable was the significantly higher percentage of damaged cells in surviving blastocysts of the C_18:0_ group (apoptotic and necrotic; 43.2 ± 20.3%) in comparison to C_18:1_ (26.0 ± 12.5%, ratio 2.27, 95% CI 2.08–2.56) and FAF SOF (26.5 ± 10.7%, ratio 2.17, 95% CI 1.96–2.38; Figure 4B).

### 3.5 Early Embryos Exposed to C_18:1_ Show Increased Cryosurvival of Blastocysts

The cryosurvival of day 8 IVP blastocysts, originating from the different FFA conditions during day 1–5 of culture, was investigated by scoring the re-inflation of the blastocoel after a 24 h revitalization culture period post-thawing. After freeze-thawing IVP blastocysts of the different groups demonstrated a striking difference in the cryosurvival rates. Blastocysts from the C_18:0_ group had a cryosurvival rate of only 17.6% (0–38.0%), which was significantly lower compared to embryos cultured in FAF SOF and C_18:1_ (OR 0.12 and 0.12, 95% CI 0.05–0.28 and 0.05–0.29, resp.; Figure 4G). Interestingly, day 8 blastocysts originating from the group exposed to FAF SOF and C_18:1_, showed a cryosurvival rate of on average 67.4% (54.6–75%) and 70.1% (33.3–100%), comparable to the cryosurvival rate of the *in vivo* derived frozen and thawed blastocysts (68.4%, Figure 4G). Furthermore, the stage of the blastocyst scored before freezing, non-expanded or expanded, had an effect on the cryosurvival rates. Expanded blastocysts cultured with C_18:1_ showed a significantly higher cryosurvival rate of 83.3% (66.7–100%), when compared to their non-expanded counterparts with a cryosurvival of 56.94% (33.3–100%; OR 5.431, 95% CI 2.06–31.53, Figure 4H). In the other groups, a similar trend was observed with the exception of blastocysts cultured in C_18:0_.

Taken together, C_18:0_ exposure during day 1–5 of IVP results in blastocysts with a low cryotolerance, demonstrated by a significantly lower cryosurvival after freeze/thawing and higher degree of cell deterioration in surviving blastocysts when compared to the other exposure groups. In contrast, embryos exposed during day 1–5 of IVP to C_18:1_ demonstrated cryosurvival rates comparable to those of *in vivo* embryos.

## 4 Discussion

A major difference between the quality of *in vivo* and *in vitro* embryos is reflected in the significantly lower cryosurvival rates of IVP embryos (Sudano et al., 2014; Moore and Hasler 2017; Viana, 2020; Ferré et al., 2020). The current study investigated the effect of low concentrations of FFA during IVP on the cryosurvival, defined as reinflation of the blastocoel after a 24 h revitalization culture period post-thawing (Dinnyés et al., 1999; Sudano et al., 2011), of IVP blastocysts. To this end, bovine embryos were *in vitro* exposed to 25 μM saturated C_18:0_ or unsaturated C_18:1_ during the first 5 days of embryo culture, resembling the physiological period that the embryo resides in the oviduct. The absence of FFA during the first 5 days of development, as reflected in the FAF SOF group, resulted in a tendency for a lower blastocyst rate on day 8 and demonstrates the importance of FFAs during this period of preimplantation development. Interestingly, blastocysts derived from the C_18:1_ group, with a significantly higher lipid content, showed a cryosurvival rate comparable with *in vivo* production MOET embryos. The cryosurvival rate of both the C_18:1_ and FAF SOF group were significantly higher than those of the C_18:0_ and standard SOF group. Embryos exposed to C_18:0_ demonstrated not only a dramatically reduced cryosurvival rate in comparison to the other groups, but the blastocysts that survived the freezing and thawing procedure also showed a significantly higher percentage of damaged cells. The current data demonstrate that exposure of embryos to unsaturated C_18:1_ significantly improved the cryosurvival rate of blastocysts, in contrast to exposure to C_18:0_. Embryo culture in FAF SOF also improved cryosurvival, but resulted in a decreased developmental potential as shown by the significantly reduced blastocystpercentage. Our results indicate that the FFA condition to which *in vitro* embryos are exposed during the first 5 days of embryo culture can have a determining role in the outcome of cryopreservation.

In analogy to the finding in this manuscript, previous studies demonstrated a clear difference in the impact of saturated and unsaturated FFAs on oocyte developmental competence. The predominating saturated FFAs in blood and follicular fluid, palmitic (C_16:0_) and C_18:0_, had a dose-dependent negative impact on the developmental competence of maturing oocytes (Leroy et al., 2005; Aardema et al., 2011; Aardema et al., 2013). Interestingly, the dominating mono-unsaturated FFA in blood and follicular fluid, oleic acid (C_18:1_), was harmless for the oocyte and could even counteract the negative impact of saturated FFAs (Aardema et al., 2011; Aardema et al., 2013). Given our new data in this study one may speculate that a similar protective effect of C_18:1_ is involved for bovine IVP embryos during day 1–5 of culture resulting in the cryoresistance properties of day 8 blastocysts.

An important key to improve the quality of IVP embryos and thus cryosurvival outcomes appears to be hidden in the oviduct, based on the outcome of the current study. Former studies have also demonstrated that embryo quality and thus the options for succesfull cryopreservation, were related to the post-fertilization conditions in which embryos reside during the early steps of development (Rizos et al., 2002; Lonergan 2007; Lonergan and Fair 2008; Gad et al., 2012). In contrast to the blastocyst yield itself, which appears to depend primarily on oocyte quality (Hendriksen et al., 2000; Lonergan et al., 2001; Dieleman et al., 2002; Rizos et al., 2002; Knijn et al., 2003; Tesfaye et al., 2007; Lonergan and Fair 2016). It is important to consider the potential impact of the addition of FFA on the genetic imprint of the embryo. The periconcenception period is a highly sensible period were epigenetic modifications can result in a lifelong impact on offspring, which was clearly demonstrated in the Dutch Hungerwinterstudies (Heijmans et al., 2008). In bovine, the relation between large offspring syndrome (LOS) and IVP embryos appears to be the most well-known example for an undesired epigenetic impact on offspring (Farin et al., 2006; Mangiavacchi et al., 2021). However, LOS and genetic aberrations in IVP embryos have primarily been related to embryo cultures with FCS. Embryo culture media without FCS, like we used in the current study, were demonstrated to result in embryos with a gene expression pattern closer to *in vivo* derived embryos (Niemann and Wrenzycki 2000; Heras et al., 2016). In the current study we exposed embryos to levels of FFA, complexed to albumin for a physiological presentation of the FFA, far below the FFA levels present in the oviduct. In an *ex-vivo* study of Jordaens et al. (Jordaens et al., 2017a), the total levels of FFA in the oviduct appeared to be comparable to the total FFA levels in blood. Based on the currently available information, we therefore expect that the low level of FFA used in the current study will have an impact on offspring like demonstrated for FCS. Nevertheless, the potential impact of FFA on the (epi)genetic imprint of the embryo certainly needs further attention in future studies.

In general, unsaturated fatty acids like C_18:1_ are shown to be less toxic on somatic cells due to their routing towards lipid droplets for storage and mitochondria for fatty acid breakdown (Cnop et al., 2001; Listenberger et al., 2001; Maedler et al., 2001; Listenberger et al., 2003; Maedler et al., 2003; Mishra and Simonson 2005). As mentioned above, *in vitro* exposure to C_18:1_ prevents the potentially toxic impact of saturated FFA on oocyte developmental competence and increased lipid uptake in cumulus cells after exposure of C_18:1_ to COCs (Aardema et al., 2011; Aardema et al., 2013; Aardema et al., 2017). Likewise the current study shows that blastocysts originating from the C_18:1_ group have a higher lipid content. Moreover, a shift towards a more unsaturated fatty acid content, like C_18:1_ and linoleic acid, in oocytes and embryos has been reported to be related to an improved quality of embryos (Kim et al., 2001; Abe et al., 2002; Haggarty et al., 2006). Therefore, the relationship between a higher lipid content in the C_18:1_ group and succesfull cryopreservation is tempting. In particular, since a high lipid content in embryos, induced by the addition of FCS, has been linked to a reduced cryopreservation and even resulted in attempts to completely remove lipid from the embryo in order to improve cryosurvival rates (Nagashima et al., 1994; Nagashima et al., 1995; Abe et al., 2002; Hara et al., 2005; Pereira et al., 2007; Sudano et al., 2011; Abe et al., 2017). Nevertheless, our current data clearly show that although an increased lipid content of embryos exposed to C_18:1_ was present, the blastocysts demonstrated significantly improved cryosurvival, i.e., comparable to the percentages of *in vivo* developed blastocysts.

In sharp contrast to the observed effect of 25 μM C_18:1_ exposure during the first 5 days of IVP on cryosurvival, C_18:0_ exposure resulted in a very low cryosurvival, and dramatically increased numbers of damaged, apoptotic and necrotic, cells in the blastocysts that survived the freezing and thawing procedures. These contrasting outcomes of saturated and unsaturated FFA are in line with previous studies in maturing oocytes (Leroy et al., 2005; Aardema et al., 2011; Aardema et al., 2017). The higher level of damaged, apoptotic and necrotic, cells in blastocysts after exposure to C_18:0_ appears to hint in the direction of apoptotic pathways. Indeed, the distinct impact of saturated and unsaturated FFA on somatic cells has for a large extent been attributed to the different routing of the fatty acids in cells. Saturated fatty acids appear to be directed towards apoptotic pathways via reactive oxygen species (ROS) and ceramide formation, and can induce mitochondrial damage and endplasmatic reticulum (ER) stress (Listenberger et al., 2001; Mu et al., 2001; Leroy et al., 2005; Wu et al., 2012; Lolicato et al., 2015; Sutton-McDowall et al., 2016; Aardema et al., 2017). Remarkably, in fresh (non-frozen) blastocysts of all groups the levels of damaged cells were low, indicating that the effect of the cryopreservation and subsequent thawing must have induced the high number of damaged cells in blastocysts from the C_18:0_ group. This finding indicates that exposure to C_18:0_ results in blastocysts that are less cryotolerant, which may ultimately result in increased embryonic death. This phenomenon certainly needs further investigation.

Besides, the toxic effects of FFA and their derived metabolites and the different incorporation into neutral lipids stored in lipid droplets of the embryo, FFA can also be incorporated into the phospholipids that reside in the membranes of the embryo. Like all biomembranes that are composed of a complex of phospholipid molecular species, gametes and embryos are also extremely sensitive to changes in temperatures (Polge 1977; Quinn 1985; Sieme et al., 2015). Lipid phase transitions have been depicted to play a major role in the cell damage that occurs in response to the chilling procedure during slow freezing, and controlling the lipid content is therefore a major step to improve the cryotolerance of embryos (Amstislavsky et al., 2019). A higher level of unsaturation and a shorter chain length of fatty acids, results in a reduced melting point and increases the fluidity and therefore flexibility of cell membranes. To this end, different fatty acids can have a very distinct effect on the cryotolerance of cells. The melting point of C_18:0_ is for example 69.6°C, and 63.1°C for C_16:0_, versus a melting point of 13.4°C for C_18:1_ and an even lower melting point for poly-unsaturated fatty acids (Stryer et al., 2002). The possibility that FFA exposure at day 1–5 of IVP is allowing C_18:1_ or C_18:0_ incorporation in phospholipids could explain the contrasting effects in increasing or decreasing cryotolerance of day 8 blastocysts. C_18:1_ incorporation into phospholipids likely prevents lipid phase segragration events much better than C_18:0_ incorporation. Indeed, in a former study the exposure of embryos to poly-unsaturated linoleic acid (C_18:2_) resulted in an increase of blastocyst cryosurvival (Hochi et al., 1999). Moreover, in a study in which ewes were fed polyunsaturated fatty acids, the oocyte quality and membrane integrity improved, while lipid phase transition temperature of the oocyte’s membranes was reduced with 11°C. This resulted in a decrease of chilling sensitivity of ewe oocytes in comparison to non C_18:2_ exposed oocytes (Zeron et al., 2002). Again supporting the possibility that the C_18:1_ versus C_18:0_ exposure during the first 5 days of IVP after incorporation in the embryos may affect membrane fluidity characteristics of day 8 IVP blastocysts.

Currently in other species, e.g., in human, embryos are routinely cryopreserved via vitrification, an ultrafast freezing method using a high amount of cryoprotectants which prevents ice crystal formation, while in the bovine species slow freezing is still routine in practice (Loutradi et al., 2008; Ferré et al., 2020). The promising results that are achieved via the vitrification process did not result in a wide use under farm conditions thus far, possibly due to the rather complicated warming procedure by an in-straw dilution (open-straw technique and potential spread of infections is not an option in bovine industry), which is required to remove the high levels of cryoprotectants before embryo transfer (Caamaño et al., 2015; Ferré et al., 2020).

To bridge the gap between *in vivo* and *in vitro* embryos, several attempts have been undertaken to mimic more closely the *in vivo* circumstances during *in vitro* embryo culture by introducing 2D and 3D culture systems with oviductal cells (Chen et al., 2017; Ferraz et al., 2017; Ferraz et al., 2018; Hamdi et al., 2019; Jordaens et al., 2020). Until now the exact lipid composition of the oviductal cells and fluid during early embryo development, which presumably has a major impact on embryo quality and thus the expected cryosurvival, has not been completely unraveled (Saint-Dizier et al., 2020). The oviductal fluid composition originates from both oviductal cell scretion (*de novo* synthesis) and blood, and includes several lipid categories; e.g., extracellular vesicles rich in phospolipids, lipoproteins, both high-density lipoproteins (HDL) and low-density (LDL) and thus cholesterol and triglycerides, and FFA (Belaz et al., 2016; Jordaens et al., 2017a; Banliat et al., 2019; Saint-Dizier et al., 2020). Apart from the total FFA levels present in the oviductal fluid that appear to reflect the total FFA composition from blood (Jordaens et al., 2017a) there is, as far as we know, no information on the composition of the lipids in the bovine oviduct.

In the current study, embryos were exposed to FFA concentrations 4–10 times below the levels that COCs are able to cope with (Leroy et al., 2005; Aardema et al., 2011; Aardema et al., 2013; Aardema et al., 2017). Embryos exposed to levels of 50 μM C_18:0_ showed a significant drop in blastocyst yield (data not shown), which suggests a high sensitivity of embryos for FFA in comparison to oocytes. This finding is in line with the results of a study in mice wherein a dramatic drop in blastocyst yield after exposing embryos to low concentrations of saturated C16:0 was demonstrated (Yousif et al., 2020). Moreover, Jordaens et al. showed that hardly any of the bovine embryos exposed to high levels of FFA, based on the metabolic stress levels in blood, developed into a blastocyst (respectively around 6 and 3%) in an insert culture system with or without the presence of oviductal cells (Jordaens et al., 2020). Interestingly, embryos appear to be more prone to the effects of FFA than the by cumulus cells enclosed oocytes, which can be explained by the protective role of cumulus cells that surround the oocyte during *in vitro* maturation, whereas embryos in conventional IVP systems lack potential protection and stabilization of the culture environment by oviductal cells. Future studies should also take into account the potential role that oviductal cells may have to protect the embryo against FFA exposure. Oviductal cells may control the exposure of metabolites to the embryo *in vivo*, in analogy with the protective and regulatory role of cumulus cells for the oocyte (Fatehi et al., 2005; Gilchrist et al., 2008; Dunning et al., 2012; Aardema et al., 2013; Lolicato et al., 2015; Aardema et al., 2017). Indeed, oviductal cells are capable to store lipids, when exposed at the apical site of the oviduct, and express the enzyme stearoyl-CoA desaturase, like cumulus cells (Aardema et al., 2017; Jordaens et al., 2017b; Aardema et al., 2018). Oviductal cells may filter and regulate the exposure of embryos to fatty acids. Thus far, it is unclear how the here used concentrations of FFA relate to the physiological concentrations of individual FFA in the oviduct during the first 5 days post-ovulation. To the best of our knowledge, there is currently no information on the specific FFA composition in the oviduct. However, the proven distinct impact of saturated versus unsaturated FFA under IVP conditions strongly suggests that the molecular species composition and total levels of FFA in the oviduct must be regulated to allow optimal embryogenesis. Likely, the FFA composition in the oviduct has a major impact on early embryonic development and information on the oviductal FFA composition could be used to optimize *in vitro* embryo culture conditions. The importance of the oviduct for early embryo development has clearly been demonstrated in studies where presumed zygotes were partly *in vivo* cultured after IVM and IVF, which resulted in increased quality and cryosurvival rates of blastocysts (Havlicek et al., 2005; Havlicek et al., 2007; Havlicek et al., 2010; Besenfelder et al., 2012). To this end, the development of systems that integrate oviductal cells during embryo culture to approach more closely the *in vivo* conditions appears to be a vital move to further improve embryo quality and cryosurvival rates (Chen et al., 2017; Ferraz et al., 2017; Ferraz et al., 2018; Hamdi et al., 2019; Jordaens et al., 2020). However, the current study lacks information on the impact of the *in vitro* FFA conditions on pregnancy outcomes after embryo transfer and offspring. Future experiments should unravel the defined physiological oviductal FFA composition, via lipidomic analysis, to which early developing embryos are exposed and the specific role that oviductal cells may play in composing the lipid profile and environment for the embryo and follow up *in vitro* experiments to investigate their impact on embryo quality and cryosurvival.

This study shows that saturated and unsaturated FFA have a very distinct impact on embryo quality and the cryotolerance of IVP blastocysts. Saturated C_18:0_ resulted in a high level of damaged cells and a low cryosurvival of blastocysts. In contrast to the high cryosurvival of embryos exposed to unsaturated C_18:1_, comparable to *in vivo* derived blastocysts. Future studies should be designed to validate these results before they can become applicable in commerciak *in vitro* bovine embryo production systems.

## Data Availability

The raw data supporting the conclusions of this article will be made available by the authors, on request.
